# Short-Term Effects of Pacifier Texture on NNS in Neurotypical Infants

**DOI:** 10.1155/2013/168459

**Published:** 2013-04-29

**Authors:** Austin L. Oder, David L. Stalling, Steven M. Barlow

**Affiliations:** ^1^Communication Neuroscience Laboratories, Department of Speech-Language-Hearing, Sciences and Disorders, University of Kansas, Room 3001, 1000 Sunnyside Avenue, Lawrence, KS 66045-7555, USA; ^2^Innara Health, Shawnee, KS 66227, USA; ^3^SPLH, Neuroscience, Human Biology and Bioengineering Director, Communication Neuroscience Laboratories, University of Kansas, Room 3001, 1000 Sunnyside Avenue, Lawrence, KS 66045-7555, USA

## Abstract

The dense representation of trigeminal mechanosensitive afferents in the lip vermilion, anterior tongue, intraoral mucosa, and temporomandibular joint allows the infant's orofacial system to encode a wide range of somatosensory experiences during the critical period associated with feed development. Our understanding of how this complex sensorium processes texture is very limited in adults, and the putative role of texture encoding in the infant is unknown. The purpose of this study was to examine the short-term effects of a novel textured pacifier experience in healthy term infants (*N* = 28). Nonnutritive suck (NNS) compression pressure waveforms were digitized in real time using a variety of custom-molded textured pacifiers varying in spatial array density of touch domes. MANCOVA, adjusted for postmenstrual age at test and sex, revealed that infants exhibited an increase in NNS burst attempts at the expense of a degraded suck burst structure with the textured pacifiers, suggesting that the suck central pattern generator (sCPG) is significantly disrupted and reorganized by this novel orocutaneous experience. The current findings provide new insight into oromotor control as a function of the oral somatosensory environment in neurotypically developing infants.

## 1. Introduction

 The human orofacial system has a remarkably rich supply of mechanoreceptors, making it one of the most sensitive tissue areas of the body in terms of tactile acuity and spatial resolution [1]. Fast-adapting type I (Meissner's corpuscles) and slow-adapting types I and II (Merkel cells and Ruffini endings, resp.) A**β** mechanoreceptors have dense innervations within perioral and intraoral structures, including the hairy skin of the face, glabrous skin of the lips, oral mucosa, and the anterior tip of the tongue. Both type I receptors—Meissner corpuscles and Merkel cells—have relatively small receptive fields with clearly defined borders and are primarily responsible for encoding tactile spatial information, including fine form and texture. Type II receptors of the face and mouth—pseudo-Ruffini endings—have larger receptive fields (<2 mm), respond to lateral skin stretch, and are presumed to function as a hybrid proprioceptor [2]. The majority of the skin of the face, lips, and oral mucosa contains slow-adapting mechanoreceptors, while the tongue tip is especially dense in fast-adapting type I mechanoreceptors, making this structure ideal for manipulation and exploration of objects in the mouth [1, 3].

The cutaneous information encoded by these mechanoreceptors is conducted along somatotopic trigeminal pathways to the brainstem, ventroposteromedial thalamus, and primary orofacial somatosensory cortex, and the strength of these signals is contingent upon the movement, force, and tissue contacts associated with the activity performed [2, 4]. It has been proposed that tactile input to the lips provides feedback that contributes to motor control during ongoing oromotor tasks in adults, including deglutition, speech, and mimicry [5–8]. Evidence of oral cutaneous sensitivity begins *in utero*, as researchers have found mouth opening at 7-8 weeks gestational age (GA) and oral reflexes at 12-13 weeks GA in response to trigeminal nerve stimulation [9–11]. Fetuses have a natural tendency to make hand to face contact early on in fetal development, and this contact is clearly visible from 12 of weeks GA [12]. Appropriate cutaneous input to the perioral and intraoral regions is essential for enhancing the skills of the growing fetus and of the newborn infant, the most crucial of which is sucking.

 The fetal suck has been observed *in utero* via ultrasound as early as 12 weeks GA [12]; however, it is typically seen between 15 and 18 weeks GA [13] and is stable by 34 weeks GA, as seen in fetal magnetoencephalography [14], and 34 weeks postmenstrual age (PMA) in healthy preterm infants [15]. During gestation, sucking is under the control of a specialized neuronal circuit known as the suck central pattern generator (sCPG), which is located in the brainstem reticular formation, and is responsible for activating groups of motoneurons that generate specific motor patterns [16–18]. One characteristic of CPGs is that they can produce a rhythmic motor pattern in the absence of sensory feedback [19]; however, the connectivity of the neuronal networks that comprise the CPGs in mammalian models and the motor signals they generate are modulated by sensory inputs [20]. Thus, afferent sensory information is important for error correction in movement or rhythmicity, as well as counteracting any unexpected perturbations in the environment [18, 19]. It has been shown that the sCPG is highly responsive to peripheral input and adapts to changes in task dynamics and local environment, such as volume and consistency of a liquid bolus or mechanical properties of a nipple [21–25]. Sensory flow from the trigeminofacial pathway also modulates the sCPG by tuning the sensitivity of orofacial reflexes [18, 26, 27], with young term infants demonstrating perioral reflex sensitivity to punctate mechanical inputs at a latency of approximately 18–21 milliseconds [28]. The sCPG is thought to regulate nonnutritive sucking (NNS) [17–19], a repetitive mouthing action on a pacifier or nipple in the absence of a liquid stimulus [29]. Therefore, many have concluded that altering the sCPG will also alter the NNS. NNS is characterized by a burst-pause pattern, with 6–12 suck cycles at a frequency of approximately 2 Hz, separated by pause “rest” periods to accommodate respiration [14, 21, 29–31]. Because coordinated NNS is necessary to produce the more difficult suck-swallow-breathe pattern associated with nutritive suck [32–35], enhancing the NNS is a logical therapeutic goal for infants with underdeveloped oromotor skills or feeding difficulties. Establishing a well-formed and patterned NNS is greatly beneficial to the developing infant, as it can improve growth and maturation, as well as gastric motility [36], stress levels [32, 33, 36–41], behavioral state [38–40, 42–45], and oral feeds [23, 24, 46, 47].

 Several studies have demonstrated the ability to modify and entrain the infant sCPG using salient patterned stimulation to the perioral and intraoral tissues, which can facilitate development and strengthen the central pathways that regulate suck [21–24, 46]; however, few studies have investigated the effects of varying physical properties of the pacifier on NNS. Early studies found that certain characteristics of a nipple or pacifier (e.g., size and shape) can influence NNS frequency [48, 49]. Recently, Zimmerman and Barlow [25] discovered that pacifier stiffness can alter the fine structure of the NNS. The present study extends these observations to consider the short-term effects of pacifier texture on ororhythmic patterning in term infants. Others have been exploring the effects of mechanical properties of feeding nipples on nutritive sucking [50], suggesting that these characteristics are important considerations during both NNS and oral feeds. A pacifier is often the most readily available and preferred form of oral stimulation for infants, both in the hospital and at home, making it one of the most significant somatic interfaces the infant will encounter. Therefore, the mechanical properties of this oral appliance are of particular interest, as they can have great consequences on the development of an infant's oromotor skills.

The aim of the present study was to examine the short-term effects of tactile domes molded onto the surface of a silicone pacifier on NNS dynamics among healthy, term infants, aged 1–4 months. Specifically, the location and spatial array density of these tactile domes were systematically varied to create 9 unique pacifier types. We hypothesized that the enhanced cutaneous salience afforded by the textured pacifiers would upregulate sCPG activity resulting in an increase in NNS burst structure and minute-rate performance measures. These data are expected to provide new information regarding cutaneous preferences of the developing orofacial system and maintenance and/or reorganization of the sCPG in neurotypical infants.

## 2. Methods

### 2.1. Participants

Participants included 28 healthy term infants (18 F, 10 M) between 1 and 4 months of postmenstrual age (PMA_mean_ at test = 50.1 weeks (SD = 3.9)) recruited from the Lawrence, KS, USA area via the Participant Recruitment and Management Core (PARC) in the Center for Biobehavioral Neurosciences in Communication Disorders (NIH P30) at the University of Kansas. The overall mean GA was 39.1 weeks, and all infants met the following inclusion criteria: born full term (>37 weeks gestation), no diagnosed medical disorders or craniofacial anomalies, and healthy at the time of testing. All infants had prior exposure to a pacifier at home, per parent report. The majority (24/28) of infants were breastfed or bottle fed with expressed breast milk, while 4 infants were bottle fed with formula. This study was approved by the Human Subjects Committee of the University of Kansas (Lawrence, KS, USA), and all parents were provided informed consent prior to the study.

### 2.2. Custom Molded Texture “Dome” Pacifiers

**Figure 1:**
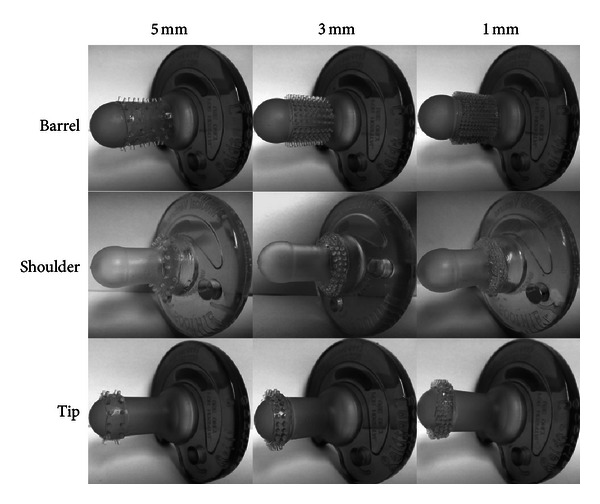
Examples of textured pacifiers. Spatial array density is represented by columns, spatial array location by rows.

The morphological features of nine different textured pacifiers, varying in spatial array density (1 mm, 3 mm, and 5 mm spacing) and location (nipple cylinder (Barrel), pacifier base (Shoulder), or the pacifier bulb (Tip)), were designed using a SolidWorks graphical model using the regular Soothie geometry as the template (see Figure 1). Subsequently, a reverse-image mold pattern was transformed into a horizontal format fabricated in 0.06 inch thick polytetrafluoroethylene (PTFE) with a small through-hole vent piercing the PTFE at the tip of each texture “dome” for captured air to escape. The resultant textured domes were approximately 1 mm in height with a 14-degree slope angle from base to tip. The template molding technique used a moisture-catalyzed medical-grade liquid silicone rubber that was pressed into the textured nodes leaving a 0.01 inch backing film to cure. After the silicone cured overnight at room temperature, the film was washed with hot water and stripped from the PTFE mold. The area of the pacifier receiving the textured film was coated lightly with the silicone gel and the textured film was brought into contact with the gel. Any excess gel was removed after air bubbles were expressed. The pacifiers were allowed to air cure overnight and were subsequently washed and sterilized with ethylene oxide (E_t_O) prior to use. The copolymerization between the Soothie pacifier and the textured film yielded textured pacifiers with excellent mechanical integrity. Each pacifier dome array type was designed to provide the infant with cutaneous stimulation localized to different soft tissue regions of the oral apparatus known to contain high densities of A**β** mechanoreceptive afferents. Overall, 9 novel pacifiers were produced (3 spatial tactile dome densities × 3 locations) [51].

### 2.3. Procedure

Participants were studied at the University of Kansas Communication Neuroscience Laboratories (CNL). Parents were asked to hold their infants in a semireclined position, while offering them a series of pacifiers, with which the infants were allowed to engage in NNS for 2 minutes each. A Nellcor OxiMAX N-600X pulse oximeter sensor was placed around each infant's wrist to monitor pulse rate and oxygen saturation (SpO_2_) throughout the study. Similar to the pacifier preference design described in the work by Zimmerman and Barlow [25], the present protocol consisted of 2-minute suck samples to assess the short-term effects of texture arrays on ororhythmic output. Four suck samples were obtained, including 3 samples with textured silicone pacifiers and 1 sample with a regular Soothie pacifier. Presentation order for the pacifiers was counterbalanced across all subjects. Each infant was randomly assigned to experience 3 textured pacifiers, where the location of the texturing was the same but the spatial arrays varied (1 mm, 3 mm, and 5 mm). Thirteen of the 28 infants participated only once, while another 13 participated twice, and 2 participated three times (in each session the infants were given pacifiers with the texturing in a different location), totaling to 45 testing sessions: 15 with Barrel texturing, 15 with Shoulder texturing, and 15 with Tip texturing (see Table 1 for demographics). Two minutes of NNS motor activity with each pacifier were collected using the NTrainer System, where automatic measures of NNS compression pressure dynamics were extracted in real time from digitized records of suck. The entire procedure lasted approximately 15 minutes. All pacifiers were gas sterilized with E_t_O prior to each use.

### 2.4. NNS Signal Analysis

Established quantitative measures of NNS dynamics were calculated for each suck sample using an automated waveform discrimination/feature detection algorithm that is implemented on the NTrainer System [23–25, 30, 31, 46]. From each 2-minute NNS sampling epoch, nipple compressions exceeding 1 cm H_2_O are automatically selected for waveform discrimination of the infant's non-nutritive suck performance. The following parameters were used as dependent variables to assess changes in the NNS pattern. (1) Total compressions indicate the sum of all pressure events per minute. (2) Non-NNS events indicate the nipple compression pressure events not associated with an NNS burst sequence. (3) NNS cycles indicate the suck compression cycles with cycle periods less than 1000 milliseconds and occurring within the NNS burst structure per minute. (4) NNS bursts indicate the sequence of two or more nipple compression cycles. (5) NNS cycles/burst indicate the mean number of NNS cycles per burst. (6) NNS cycles % total indicate the NNS cycles expressed as a percentage of total nipple compressions ([Burst-related NNS cycles/Total Mouthing Events] × 100). (7) Pressure amplitude indicates the average amplitude of pressure (in cmH_2_0) generated by infants across each epoch. (8) NNS spatiotemporal index (STI) indicates the cumulative sum of the standard deviations of a set of NNS pressure trajectories. The NNS STI applies time and amplitude normalization on suck compression signals associated with the NNS burst and calculates the cumulative sum of the standard deviations (indexed at 100 ms intervals) to yield the NNS STI value. In general, as the NNS burst structure develops, the STI values decrease from 90 (disorganized suck pattern) to approximately 35 (highly organized and developed suck pattern). This captures the gestalt of central pattern generation for NNS [24, 46]. A multivariate analysis of covariance (SPSS version 20.0), controlling for age at the time of testing and sex, was performed to examine NNS outcome measures as a result of the texture location, texture density, and the presence of a textured versus a smooth pacifier.

## 3. Results

A multivariate analysis of covariance (MANCOVA) with the 8 NNS parameters as dependent variables and texture location and texture array density as independent variables revealed no statistically significant differences on any of the NNS outcome measures, when controlling for PMA at the time of testing and sex (see Table 2). Therefore, we chose to examine whether texturing of any type, regardless of location or spacing of the tactile domes, had an effect on NNS. All NNS recordings obtained with any textured pacifier were pooled and analyzed as a general textured condition. MANCOVA revealed statistically significant differences between NNS outcomes in the textured versus the control smooth pacifiers (Pillai's trace = 0.122, *F*(7,166) = 3.291, *P* = 0.003, multivariate *η* ^2^= 0.122), adjusted for PMA at the time of testing and sex. Seven of the 8 NNS parameters were significantly different between the two pacifier conditions, when evaluating the effects at an alpha level of 0.05 (Table 3).

Overall, infants displayed a significantly greater number of total compressions, NNS cycles, NNS cycles per burst, and NNS cycles as a percentage of the total nipple compressions, as well as a higher compression pressure amplitude and lower STI with the smooth pacifier than with the textured pacifiers. These findings suggest that the regular Soothie pacifier is preferred over the novel textured pacifiers utilized in this study by infants (1–4 months of age) as manifested in patterned, stable NNS.

## 4. Discussion

In the present study, we have demonstrated that neurotypical infants significantly reorganize their NNS output in the presence of pacifiers with custom-molded tactile domes varying in spatial array density and location. The time scale for this oromotor reorganization in the presence of a textured pacifier is rapid and sustained during the 2-minute sampling epochs. The age range studied (1–4 of months postnatal age) covers a developmental stage in which many neuronal circuits have already been formed [52], but appropriate sensory experiences are necessary during this critical period of oral sensorimotor development to further strengthen and regulate these pathways [23, 53]. 

 NNS is a complex behavior and shows characteristic developmental patterns, increasing in complexity and organization with advancing maturity [54]. It is a fine oromotor behavior that affords the infant readiness skills to produce the more challenging suck-swallow-breathe pattern associated with nutritive sucking [32–35]. This sensorimotor progression corresponds to the increasing coordination of interconnected CPGs that mediate sucking, swallowing and breathing [19, 55–57] and indicates sensory and motor system integrity and positive neurodevelopment [58]. For this reason, NNS is often targeted in therapy for infants with an uncoordinated suck and/or feeding difficulties. 

 Sensory experiences functionally alter an infant's brain. As sensory signals proliferate, a cascade of cellular and molecular processes alters neurochemistry and brain structure [59]. Early orosensory experiences are critical, as they prepare the infant for a wide range of sensorimotor actions that ultimately support suck, feeding, airway protection, and state control [19]. The study presented here demonstrates that the novel experience afforded by the tactile domes is not preferred over that provided by the smooth pacifier in a short-term sampling paradigm.

 Of the 8 NNS parameters, four (NNS cycles, NNS bursts, NNS cycles/burst, and pressure amplitude) were significantly different at a very conservative alpha level (*P* ≤ 0.006). NNS cycles, NNS cycles/burst, and the mean pressure amplitude were significantly decreased with the use of the textured pacifiers, while NNS bursts were increased. It is unclear as to why the average number of NNS bursts was increased while all other aspects of the suck were decreased. Perhaps the number of bursts—defined as any sequence of two or more nipple compression cycles greater than 1 cmH_2_O in amplitude—was increased because the average number of cycles within each burst (NNS cycles/burst) was decreased, allowing infants to produce more bursts that contain less suck cycles within the 2-minute samples. Another possibility is that the sCPG was undergoing resets due to the high salience of the textured pacifiers.

**Figure 2:**
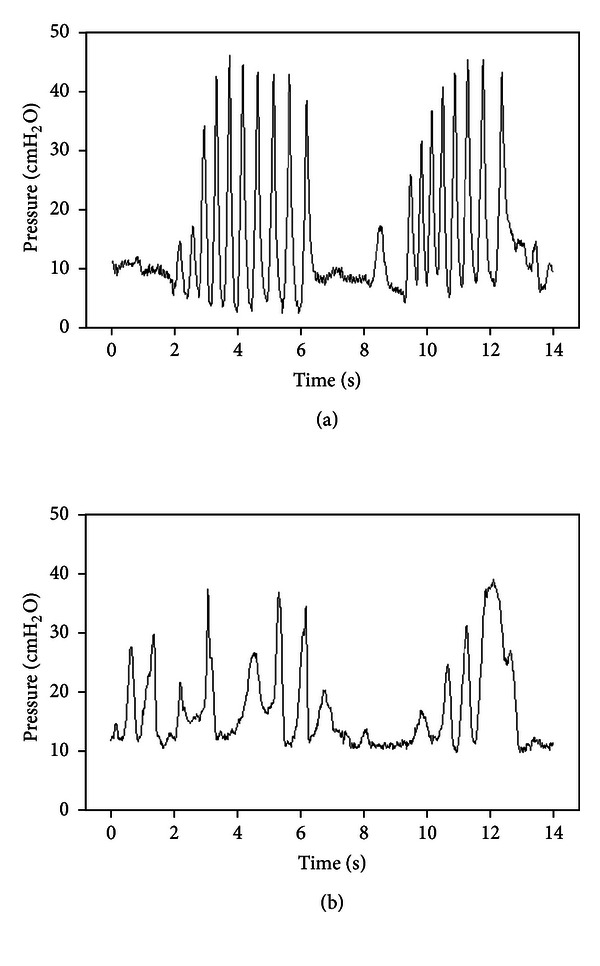
(a) Characteristic burst-pause pattern of NNS of an infant. (b) Oral compression pattern of the same infant when given the 1 mm textured Barrel array pacifier.

 The following are possible explanations as to why the textured pacifiers significantly decreased nearly all other NNS parameters in healthy term infants. It may be that during the use of the textured Barrel and Shoulder pacifiers, the infants were unable to form a tight oral seal around the base, as the tactile domes may have prevented airtight closure of the lips onto the pacifiers. This would significantly decrease the negative intraoral pressure necessary to produce the suck, causing a decrease in oromotor activity. For many infants, the presence of the textured surfaces inside the mouth elicited a “chewing” response. According to parent report, none of the infants enrolled in this study were teething at the time of testing. Thus, it is speculated that the textured Barrel and Tip pacifiers offered stimulation to the gums that encouraged the infants to chew with their gums on the textured pacifiers. This type of activity would also significantly alter the NNS records, resulting in motor output that does not resemble NNS patterning, but rather chewing (see Figure 2). Perhaps the high salience of the tactile domes would be more appropriate for a population in which the elicitation of chewing is desirable, such as individuals with feeding problems following a stroke.

 The conversion of suck to a bite/chew manifest by infants in this study using the textured “dome” pacifiers was an unexpected and remarkable finding. This effect on oromotor patterning has potential neurotherapeutic applications for older children and adults who exhibit feeding disorders following brainstem and/or cerebral stroke. The development of a textured “dome” appliance that is pneumatically-charged to generate physiologically relevant patterns of somatosensory experience may be useful in older patients to facilitate the recovery of masticatory oromotor patterning in transition to attainment of feeding skills. 

 The present results also reinforce the potent effect of the local sensory environment in modulating the output of the sCPG in young infants. The somatosensory channel, mediated via trigeminal primary afferents (V2 maxillary division and V3 mandibular division), has been shown to modulate suck in infants following changes in pacifier stiffness [25] and during the presentation of pulsed pneumatic orocutaneous inputs in term [22] and preterm infants [23, 24, 46]. This study is the first study to examine the short-term effects of pacifier texture on NNS and the sCPG. Additional studies are needed to examine the long-term effects of repeated exposures to textured stimuli at various spatial array frequencies on ororhythmic output, which could potentially influence firing patterns of orofacial lower motor neurons [23, 24, 46].

## 5. Conclusion

 In summary, the present investigation revealed that healthy term infants modify the spatiotemporal properties of the NNS central pattern generator when presented with a novel orosensory experience. Infants demonstrated an overall decrease in ororhythmic output, suggesting that a textured surface on a pacifier (regardless of spatial array and location) is not beneficial to oromotor development. The physical properties of a pacifier are of great importance, given that it is the most common interface to an infant during oral stimulation. Our results provide further evidence of the potent effect of the local sensory environment in modulating the pattern production of the sCPG in infancy. 

## Figures and Tables

**Table 1:** Characteristics of study infants.

	Barrel (*N* = 15)	Shoulder (*N* = 15)	Tip (*N* = 15)
Gender (♂ : ♀)	6 : 9	7 : 8	4 : 11
GA_birth_ (weeks)	39.18 (1.21)	39.04 (1.26)	39.14 (1.2)
PMA_test_ (weeks)	51.91 (3.68)	49.37 (3.91)	48.98 (3.54)

**Table 2:** Mean and standard deviation for all measured variables across all pacifiers and tactile dome arrays.

	Barrel			Shoulder			Tip			Smooth
	5 mm	3 mm	1 mm	5 mm	3 mm	1 mm	5 mm	3 mm	1 mm	—
Total compressions	197.4 (35.27)	196.47 (29.53)	190.47 (26.76)	207.57 (37.58)	210.14 (31.55)	195.71 (27.9)	196.93 (48.29)	189.53 (35.53)	187.29 (33.42)	212.64 (44.29)
Non-NNS events	21.67 (16.29)	23.93 (27.49)	16.27 (9.79)	16.64 (10.78)	21.93 (14.24)	17.93 (16.31)	26.2 (18.1)	18.8 (12.0)	20.86 (13.52)	16.6 (21.41)
NNS cycles	87.87 (17.46)	86.27 (17.92)	87.1 (13.33)	95.46 (20.28)	94.11 (16.2)	88.89 (18.71)	85.37 (27.19)	85.37 (20.49)	83.21 (17.69)	98.02 (18.0)
NNS bursts	19.27 (5.7)	16.67 (4.7)	16.33 (5.15)	16.71 (6.65)	18.57 (6.73)	16.29 (5.5)	19.73 (5.91)	16.8 (4.72)	19.07 (5.77)	15.09 (4.97)
NNS cycles/burst	9.6 (5.29)	10.8 (5.72)	11.4 (5.97)	13.79 (9.34)	11.5 (6.75)	12.43 (8.17)	10.4 (10.03)	10.67 (6.3)	8.93 (4.41)	15.2 (12.19)
NNS cycles % total	89.11 (7.54)	87.86 (12.54)	91.4 (5.04)	91.59 (5.84)	89.46 (6.55)	90.17 (9.81)	85.79 (10.64)	89.37 (7.6)	88.49 (7.38)	92.49 (6.11)
Pressure amplitude	17.05 (10.2)	19.05 (12.08)	17.81 (7.9)	20.03 (7.42)	16.78 (6.28)	17.44 (6.95)	15.19 (11.55)	15.4 (8.68)	18.6 (13.11)	23.13 (7.76)
STI	74.43 (8.16)	74.52 (6.12)	75.26 (4.88)	72.72 (7.91)	71.25 (10.78)	71.52 (9.23)	77.1 (5.6)	71.63 (6.57)	73.27 (6.75)	70.06 (10.44)

**Table 3:** Descriptive statistics for all measured variables when pooled across all textured pacifiers (*P* < 0.05).

	Textured	Smooth	*F*	Sig.	*η*²
Total compressions	196.73 (34.27)	212.64 (44.29)	6.561	**0.011**	0.037
Non-NNS events	20.2 (16.06)	16.6 (21.41)	1.617	0.205	0.009
NNS cycles	88.12 (15.96)	98.02 (18.0)	9.979	**0.002**	0.055
NNS bursts	17.72 (5.66)	15.09 (4.97)	7.745	**0.006**	0.043
NNS cycles/burst	11.04 (7.02)	15.2 (12.19)	7.875	**0.006**	0.044
NNS cycles % total	89.23 (8.36)	92.49 (6.11)	5.779	**0.017**	0.033
Pressure amplitude	17.45 (9.48)	23.13 (7.76)	14.009	**0.000**	0.075
STI	73.56 (7.5)	70.06 (10.44)	5.816	**0.017**	0.033
